# Prevalence of Ruptured Intracranial Aneurysms in a Tertiary Care Hospital of Nepal

**DOI:** 10.31729/jnma.4328

**Published:** 2019-06-30

**Authors:** Suraj Thulung, Binit Aryal, Aashish Baniya, Kajan Ranabhat, Bibhusan Kalu Shrestha

**Affiliations:** 1 Department of Neurosurgery, Upendra Devkota Memorial National Institute of Neurological and Allied Sciences, Bansbari, Kathmandu, Nepal; 2 Department of Radiology, Upendra Devkota Memorial National Institute of Neurological and Allied Sciences, Bansbari, Kathmandu, Nepal

**Keywords:** *Computed tomography angiogram*, *intracranial aneurysm*, *subarachnoid hemorrhage*

## Abstract

**Introduction:**

Intracranial aneurysms affect 3-8 percent of the world's population, with ruptured aneurysms being the most common cause of subarachnoid hemorrhage. The sensitivity of Computed Tomography Angiogram in diagnosing intracranial aneurysm is 97%. The aim of our study is to find out the prevalence of ruptured intracranial aneurysms among all the admitted cases encountered in our hospital.

**Methods:**

A descriptive cross-sectional study was done at Upendra Devkota Memorial National Institute of Neurological and Allied Sciences from 2016 to 2018. Convenience sampling method was done. In order to detect the site and size of aneurysms, 16 slice Siemens Computed Tomography with Computed Tomography angiogram was used. Ethical approval was obtained from the Institutional Review Committee at Upendra Devkota Memorial National Institute of Neurological and Allied Sciences. Based on demographic data and computed tomography angiography findings, various morphometric parameters along with demographic parameters were considered for the study.

**Results:**

Among 10,856 cases, prevalence of ruptured intracranial aneurysms were found in 42 (0.386%) [Confidence Interval= 0.395 to 0.377]. Among 42 cases, Middle Cerebral Artery aneurysm was present on 16 (39.02%) followed by Anterior Communicating Artery on 14 (34.14%), then Posterior Communicating Artery on 5 (12.19%). The largest neck and dome size were seen in basilar tip aneurysm with size of 11mm and 8mm respectively. The most common type was Fischer grade 4.

**Conclusions:**

The prevalence of ruptured intracranial aneursyms were found to be higher as compared to the other international studies.

## INTRODUCTION

Sub Arachnoid Hemorrhage (SAH) is defined as the presence of blood in the sub arachnoid spaces which is life threatening condition. 80 to 90 % of sub arachnoid bleed is attributed to rupture of cerebral aneurysm. Non-contrast computed tomography (CT) of brain is the initial imaging modality of choice for the diagnosis of sub arachnoid hemorrhage.^1,2^ Further evaluations with Computed Tomography Angiogram (CTA) or Cerebral angiography is required when the presence of SAH is established. Non-contrast CT followed by CTA diagnoses aneurismal SAH in 99% of the cases.^3,4^ If the non contrast CT scan is normal, lumbar puncture remains necessary to avoid potential misdiagnosis.^2–5–6^ Fisher et al. have developed a reliable system for grading of ruptured aneurysm.^7^ There are very few data on brain aneurysm, its location, size, type and grade among Nepalese populations. There is increasing trend of diagnosis of cerebral aneurysm by recent diagnostic modalities.

The aim of this study is to find out the prevalence of ruptured intracranial aneurysm among all the admitted cases in our hospital.

## METHODS

This is a descriptive cross-sectional study done at Upendra Devkota Memorial National Institute of Neurological and Allied Sciences (UDM-NINAS). Ethical approval was obtained from the Institutional Review Committee at UDM-NINAS.


$$\text{n}={\text{Z}}^{2}\times (\text{p}\times \text{q})/{\text{d}}^{2}$$

where,

n = sample size
p = prevalence, 50%
q = 1-p
d = margin of error, 1%
Z = 1.96 at 95% CI

Calculated sample size was 9604. Taking non response rate of 10%, sample size is 10565. Convenience sampling was done. We retrieved data from hospital medical records of UDM-NINAS from 2016 to 2018. Demographic details like age, sex, location and morphometric details like aneurysmal neck size, dome size and fisher grading were considered. All patients underwent CT and CT Angiogram in 16 slice scanner. SAH was evaluated in non-contrast CT head using window level of 40 and width 120. Angiograms were evaluated using MIP images (W.872 level 305) and 3D reconstruction/MDR images. In this study, patient with ruptured aneurysm with sub arachnoid hemorrhage were included. Since all of the admitted cases were studied from the last two years, whole sampling method was applied. Reporting bias from the radiologist can always be present and leads to false data. Missing of records can effect in actual prevalence of aneurysm and its types and size. Aneurysm's location, size, mean neck size, mean dome size and Fischer grade were observed. Data were analyzed in SPSS version 23.

## RESULTS

**Figure 1.**
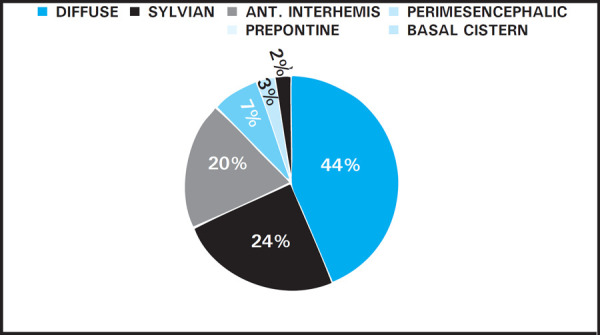
Distribution of SAH.

Among all the admitted cases in the last two years (10,856), the prevalence of ruptured intracranial aneurysm was found to be 0.38% (42) [Confidence Interval= 0.395 to 0.377]. In two year, we have encountered total 42 cases of ruptured aneurysm. The mean age of aneurysm rupture was found to be 56.19 years ± 13.011. There was female preponderance (78.04%) with male to female ratio of 1: 3.27. The three most common locations of SAH were diffuse (43.9%), sylvian (24.39%) and anterior inter-hemispheric (19.51%) (Figure 1).

**Figure 2.**
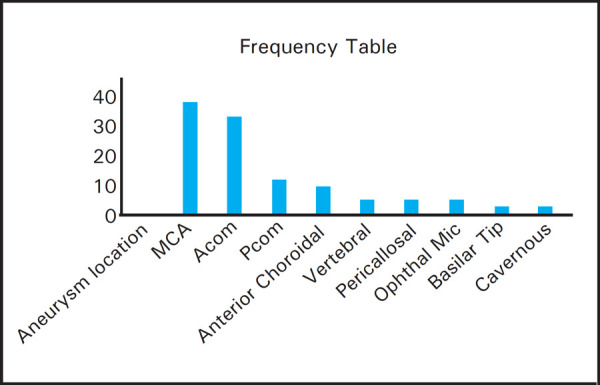
Frequency distribution of aneurysm location.

Concerning Fischer grade, grade 4 (39.02%) was most commonly encountered. The most common location of ruptured aneurysm was MCA (39.02%) followed by ACOM (31.14%) and PCOM (12.19%) respectively (Figure 2). There were 5 cases of multiple intracranial aneurysms, with notable association of multiplicity seen in MCA territory aneurysms.

**Figure 3.**
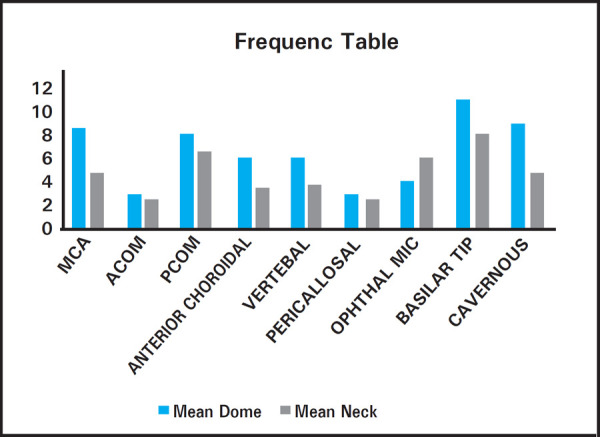
Frequency distribution of mean dome and mean neck size of aneurysm at various locations.

Upon morphometric analysis, we found larger sized aneurysm mostly to be in posterior circulation especially at and around basilar tip, having mean dome diameter of 11 mm followed by cavernous (9 mm) and MCA (8.5 mm). Similarly, considering mean neck diameter, larger neck size was found in basilar tip (8 mm) followed by PCOM (6.5 mm), and Ophthalmic segment of ICA (6.1 mm) (Figure 3).

## DISCUSSION

Considerable variation exists in the annual incidence of aneurysmal SAH in different regions of the world. A World Health Organization study established a 10-fold variation in the age adjusted annual incidence in countries of Europe and Asia.^8^

In our study, we found aneurysm rupture was more common at the mean age of 56.19± 13.011 years. This data is consistent with studies that showed that the incidence of aneurysmal SAH increases with age, with a typical average age of onset in adults of 50 years of age.^9,10,11^

The study conducted by Ghods AJ. et al. showed that SAH)occur more often in women and are thought to be a result of both hormonal influences and variation in wall shear stress, which is congruent with the result of our study (M:F = 1:3.272).^12^

Among these 42 cases, the most common distribution of Sub-arachnoid hemorrhage noted is diffuse 18 (43.9%) type. Other locations commonly noted were along the sylvian fissure 10 (24.39%) and anterior inter-hemispheric fissure 8 (19.51%). Considering Fischer grading for segregation of sub-arachnoid hemorrhage, most common grade noted was Fisher grade 4, in 16 (39.02%) cases followed by Fisher grade 3 and 2, in 10 (24.39%) and then by Fisher grade 1, in 5 (11.9%) cases.

We observed that the most common location of aneurysm was in the branches of middle cerebral artery followed by anterior communicating and posterior communicating artery respectively. Contrary to our findings, Forget and Colleagues reported the most frequent site of aneurysm rupture was anterior communicating artery (29%) followed by posterior communicating (19.6%) then by basilar (14.7%) and then only by middle cerebral artery (11.8%).^13^ However in the study cohort of 1993 patients (61% women) with saccular ruptured intracranial aneurysms, middle cerebral (32%) and anterior communicating artery (32%) were reported to have highest incidence of aneurysm followed by posterior communicating arteries respectively (14%).^14^

Upon morphometric study in our series of 42 cases, largest dome of aneurysm was seen basilar tip (11mm) followed by cavernous (9mm) and MCA (8.5mm). Largest neck diameter of aneurysm was noted in basilar tip followed by posterior communicating and ophthalmic segment aneurysm. The mean diameter of dome and neck in our observations were 6.53mm and 4.80mm respectively, which appeared to be of smaller size than the reported mean size of ruptured intracranial aneurysm, 8mm, in a study done by Weir B. et al.^15^

Jeong YJ et al. reported in his study that the critical sizes at which the incidence of rupture increases for aneurysms are from 4 to 10 mm.^16^ Ohashi et al. reported that ruptured ACA aneurysms were significantly smaller (6.6 mm) than ICA or MCA aneurysms (8.5 mm and 8 mm).^17^ This result is congruent with the mean dome size of ruptured MCA and ACom aneurysm in our study. Unlike our results, Qureshi et al. reported MCA aneurysms to be of larger size than aneurysms at other location.^18^

The limitations of this study are that the study site is one of the few tertiary hospital for neurosurgery care which might have increased the prevalence. Since this study is only conducted in only one tertiary centre, the results may not be generalized in other centers.

## CONCLUSIONS

The prevalence of the ruptured intracranial aneurysm in tertiary hospital was found to be higher as compared to other studies done in other countries. Multi-centre studies are encouraged to increase the scientific quality of the findings, and better study design could be applied.

## Conflict of Interest

**None.**
